# Supplementary material for: The oligomeric assembly of galectin-11 is critical for anti-parasitic activity in sheep (Ovis aries)
Source: Commun Biol. 2020 Aug 21;3:464. doi: 10.1038/s42003-020-01179-7 (PMC7442640; doi:10.1038/s42003-020-01179-7)
Supplement: Supplementary file 1 — Supplementary Information [file 42003_2020_1179_MOESM1_ESM.pdf]

Supporting Information

Supplementary Table 1. Hydrodynamic properties of LGALS-11 proteins

**LGALS-11**	Oligomeric species	S^{o}_{20},w[1] (Svedberg, S)	f/f0[2]
Isoform 1	Tetramer	4.0	1.30
Isoform 2	Monomer	1.6	0.56
Isoform 2	Dimer	2.5	0.89
DI-m[2]	Monomer	1.6	1.1
DI-m	Dimer	2.5	1.7

[1] Standardised sedimentation coefficients obtained from the ordinate maxima of the *c(s)* distribution peaks calculated at a concentration of 0.10 mg/ml of LGALS-11 isoform 1. For isoform 2 the *P*-value decreased from 0.95 to 0.0 to allow determination of the ordinate maxima of distinct *c(s)* distribution peaks with baseline. [2] Recombinant dimerisation mutant (DI-m) protein.
