# Supplementary material for: The oligomeric assembly of galectin-11 is critical for anti-parasitic activity in sheep (Ovis aries)
Source: Commun Biol. 2020 Aug 21;3:464. doi: 10.1038/s42003-020-01179-7 (PMC7442640; doi:10.1038/s42003-020-01179-7)
Supplement: Supplementary file 2 — Description of Additional Supplementary Files [file 42003_2020_1179_MOESM2_ESM.pdf]

Description of Additional Supplementary Files

File Name: **Supplementary Data 1**
Description: Source for fig 4a.
