# Supplementary material for: The oligomeric assembly of galectin-11 is critical for anti-parasitic activity in sheep (Ovis aries)
Source: Commun Biol. 2020 Aug 21;3:464. doi: 10.1038/s42003-020-01179-7 (PMC7442640; doi:10.1038/s42003-020-01179-7)
Supplement: Supplementary file 4 — Reporting Summary [file 42003_2020_1179_MOESM4_ESM.pdf]

nature research

Corresponding author(s): COMMSBIO-19-1639, Travis Beddoe

Last updated by author(s): Jun 26, 2020

Reporting Summary

Nature Research wishes to improve the reproducibility of the work that we publish. This form provides structure for consistency and transparency in reporting. For further information on Nature Research policies, see Authors & Referees and the Editorial Policy Checklist.

Statistics

For all statistical analyses, confirm that the following items are present in the figure legend, table legend, main text, or Methods section.

n/a	Confirmed	
☐	☒	The exact sample size (*n*) for each experimental group/condition, given as a discrete number and unit of measurement
☐	☒	A statement on whether measurements were taken from distinct samples or whether the same sample was measured repeatedly
☒	☐	The statistical test(s) used AND whether they are one- or two-sided<br>*Only common tests should be described solely by name; describe more complex techniques in the Methods section.*
☒	☐	A description of all covariates tested
☒	☐	A description of any assumptions or corrections, such as tests of normality and adjustment for multiple comparisons
☒	☐	A full description of the statistical parameters including central tendency (e.g. means) or other basic estimates (e.g. regression coefficient) AND variation (e.g. standard deviation) or associated estimates of uncertainty (e.g. confidence intervals)
☒	☐	For null hypothesis testing, the test statistic (e.g. *F*, *t*, *r*) with confidence intervals, effect sizes, degrees of freedom and *P* value noted<br>*Give P values as exact values whenever suitable.*
☒	☐	For Bayesian analysis, information on the choice of priors and Markov chain Monte Carlo settings
☒	☐	For hierarchical and complex designs, identification of the appropriate level for tests and full reporting of outcomes
☒	☐	Estimates of effect sizes (e.g. Cohen's *d*, Pearson's *r*), indicating how they were calculated

Our web collection on statistics for biologists contains articles on many of the points above.

Software and code

Policy information about availability of computer code

Data collection	CCP4 program suite
Data analysis	SEDFIT program was used to fit AUC data; PHENIX and COOT for cystallagraphy

For manuscripts utilizing custom algorithms or software that are central to the research but not yet described in published literature, software must be made available to editors/reviewers. We strongly encourage code deposition in a community repository (e.g. GitHub). See the Nature Research guidelines for submitting code & software for further information.

Data

Policy information about availability of data

All manuscripts must include a data availability statement. This statement should provide the following information, where applicable:

- Accession codes, unique identifiers, or web links for publicly available datasets
- A list of figures that have associated raw data
- A description of any restrictions on data availability

The information supporting the conclusions of this article is included in the article. The atomic coordinates and observed structural factors have been deposited (under codes 6N3R and 6N44, respectively) in the Research Collaboratory for Structural Bioinformatics PDB. The LGALS-11 sequences (accession nos. MH069213 and MH069214) representing isoforms 1 and 2, respectively, were deposited in the NCBI database. The authors declare that the data supporting the findings of this study available within the paper, any other relevant data in this study available from the corresponding authors on reasonable request.

Field-specific reporting

Please select the one below that is the best fit for your research. If you are not sure, read the appropriate sections before making your selection.

☒ Life sciences ☐ Behavioural & social sciences ☐ Ecological, evolutionary & environmental sciences

For a reference copy of the document with all sections, see nature.com/documents/nr-reporting-summary-flat.pdf

Life sciences study design

All studies must disclose on these points even when the disclosure is negative.

Sample size	16 sheep were sequenced due to limit of samples available
Data exclusions	no data exclusion
Replication	All experiments were performed in duplicate or triplicate
Randomization	not applicable
Blinding	not applicable

Reporting for specific materials, systems and methods

We require information from authors about some types of materials, experimental systems and methods used in many studies. Here, indicate whether each material, system or method listed is relevant to your study. If you are not sure if a list item applies to your research, read the appropriate section before selecting a response.

Materials & experimental systems

n/a	Involved in the study	
☒	☐	Antibodies
☒	☐	Eukaryotic cell lines
☒	☐	Palaeontology
☐	☒	Animals and other organisms
☒	☐	Human research participants
☒	☐	Clinical data

Methods

n/a	Involved in the study	
☒	☐	ChIP-seq
☒	☐	Flow cytometry
☒	☐	MRI-based neuroimaging

Antibodies

Antibodies used	no antibodies used in this study
Validation	no antibodies used in this study

Animals and other organisms

Policy information about studies involving animals; ARRIVE guidelines recommended for reporting animal research

Laboratory animals	Study did not involve laboratory animals
Wild animals	Study did not involve wild animals
Field-collected samples	Abomasum samples were collected from sheep raised under normal farming practice in Australia
Ethics oversight	Handling of animals and experimental procedures were approved by the Monash University Animal Ethics Committee (Ethics # SOBSA/P/2009/44)

Note that full information on the approval of the study protocol must also be provided in the manuscript.
